# Elongation Factor Thermo Unstable (EF-Tu) Moonlights as an Adhesin on the Surface of *Mycoplasma hyopneumoniae* by Binding to Fibronectin

**DOI:** 10.3389/fmicb.2018.00974

**Published:** 2018-05-15

**Authors:** Yanfei Yu, Hongen Wang, Jia Wang, Zhixin Feng, Meng Wu, Beibei Liu, Jiuqing Xin, Qiyan Xiong, Maojun Liu, Guoqing Shao

**Affiliations:** ^1^Key Laboratory of Veterinary Biological Engineering and Technology of Ministry of Agriculture, Institute of Veterinary Medicine & National Center for Engineering Research of Veterinary Bio-products, Jiangsu Academy of Agricultural Sciences, Nanjing, China; ^2^College of Animal Science and Technology, Shanxi Agricultural University, Taigu, China; ^3^National Contagious Bovine Pleuropneumonia Reference Laboratory, Division of Bacterial Diseases, State Key Laboratory of Veterinary Biotechnology, Harbin Veterinary Research Institute of Chinese Academy of Agricultural Sciences, Harbin, China; ^4^Key Lab of Food Quality and Safety of Jiangsu Province-State Key Laboratory Breeding Base, Nanjing, China

**Keywords:** *Mycoplasma hyopneumoniae*, swine tracheal epithelial cells (STEC), adherence, EF-Tu, fibronectin

## Abstract

*Mycoplasma hyopneumoniae* is a colonizing respiratory pathogen that can cause great economic losses to the pig industry worldwide. Although putative virulence factors have been reported, the pathogenesis of this species remains unclear. Here, we used the virulent *M. hyopneumoniae* strain 168 to infect swine tracheal epithelial cells (STEC) to identify the infection-associated factors by two-dimensional electrophoresis (2-DE). Whole proteins of *M. hyopneumoniae* were obtained and compared with samples cultured in broth. Six differentially expressed proteins with an increase in abundance of ≥1.5 in the cell infection group were successfully identified. A String network of virulence-associated proteins showed that all the six differential abundance proteins were involved in virulence of *M. hyopneumoniae*. One of the most important upregulated hubs in this network, elongation factor thermo unstable (EF-Tu), which showed a relatively higher expression in *M. hyopneumoniae*-infected STEC and obtained a higher score on mass spectrometry was successfully recombined. In addition to its canonical enzymatic activities in protein synthesis, EF-Tu was also reported to be located on the cell surface as an important adhesin in many other pathogens. The cell surface location of EF-Tu was then observed in *M. hyopneumoniae* with flow cytometry. Recombinant EF-Tu (rEF-Tu) was found to be able to adhere to STEC and anti-rEF-Tu antibody enclosed *M. hyopneumoniae* decreased adherence to STEC. In addition, surface plasmon resonance (SPR) analysis showed that rEF-Tu could bind to fibronectin with a specific and moderately strong interaction, a dissociation constant (*K*_D_) of 605 nM. Furthermore, the block of fibronectin in STEC also decreased the binding of *M. hyopneumoniae* to the cell surface. Collectively, these data imply EF-Tu as an important adhesin of *M. hyopneumoniae* and fibronectin as an indispensable receptor on STEC. The binding between EF-Tu with fibronectin contributes to the adhesion of *M. hyopneumoniae* to STEC.

**HIGHLIGHTS**

- Elongation factor thermo unstable (EF-Tu) exists on the cell surface of *M. hyopneumoniae*.
- EF-Tu moonlights as an adhesin of *M. hyopneumoniae*.
- The adhesive effect of EF-Tu is partly meditated by fibronectin.

## Introduction

Respiratory diseases are among the most important health problems associated with swine production. *Mycoplasma hyopneumoniae* is the primary pathogen responsible for swine enzootic pneumonia. This infection is highly prevalent (ranging between 38 and 100%) in almost all areas of pig production worldwide, and causes significant economic losses (Thacker and Minion, 2010).

Though a few researches found *M. hyopneumoniae* invasive, it is mostly considered that *M. hyopneumoniae* is an extracellular pathogen. It predominantly colonizes and destroys the epithelial surfaces of the respiratory tract (DeBey and Ross, 1994). Adhesion along the entire length of the respiratory epithelium is recognized as the first and most important step in *M. hyopneumoniae* colonization and infection (Thacker and Minion, 2010; Maes et al., 2017). Several proteins have been identified to be involved in adherence. P97 was the first characterized adhesin of *M. hyopneumoniae*. It binds to the cilia of respiratory epithelial cells by the C-terminal portion, identified as R1 (Zhang et al., 1995). However, other factors or additional proteins may also contribute to adherence. For example, the P102 protein, which is located in the same operon as P97, also participates in virulence as it is expressed *in vivo* during infection (Adams et al., 2005) and can recruit plasminogen and fibronectin to the surface of *M. hyopneumoniae* (Seymour et al., 2012). In addition to these findings, factors such as P159 (Burnett et al., 2006), P146 (Mayor et al., 2007), P216 (Wilton et al., 2009), Mhp271 (Deutscher et al., 2012), Mhp107 (Seymour et al., 2011), and Mhp683 (Bogema et al., 2011) have also been shown to be associated with the adhesion process. However, the pathogenesis and possible virulence factors of *M. hyopneumoniae* are not yet fully known (Simionatto et al., 2013), and the exact mechanism by which it adheres to epithelial cells and a clear picture of its virulence and pathogenicity remain to be understood.

The comparative proteomics analysis presented here demonstrated a comprehensive and proteome-wide approach to identify novel proteins and their interaction involved in the virulence of *M. hyopneumoniae* in swine tracheal epithelial cells (STEC), which is one of the target tissues of *M. hyopneumoniae*. Importantly, the results of this study could facilitate uncovering new biological activities or unknown functions of known proteins that can lead to a more complete understanding of the virulence-associated proteins and the complex biological and infectious processes taking place during infection. For example, the canonical function of the cytoplasmic protein, EF-Tu is acting as a GTPase on the binding of aminoacyl-tRNA to the ribosome-mRNA complex during translation elongation in protein synthesis (Voorhees and Ramakrishnan, 2013). However, in addition to its conserved activities in cytoplasm, EF-Tu can also be expressed on the cell surface as an important adhesin in numerous pathogens, such as *Mycobacterium leprae* (Marques et al., 1998), *M. pneumoniae* (Dallo et al., 2002), *Streptococcus suis* (Li et al., 2015), and so on. In this study, the non-canonical function, the pathogenic role of the surface protein EF-Tu in *M. hyopneumoniae* will be explored.

## Materials and methods

### Ethics statements

All animal experiments were performed in Jiangsu Academy of Agricultural Sciences with the approval of the Committee on the Ethics of Animal Experiments of (JAAS no. 20141107). All experimental procedures conformed to the guidelines of Jiangsu Province Animal Regulations (Government Decree No. 45) in accordance with international law.

### Bacterial strains and growth conditions

*M. hyopneumoniae* strain 168 (GenBank accession CP002274) was originally isolated in 1974 from an Er-hua-lian pig (a local Chinese breed that is very sensitive to *M. hyopneumoniae*) exhibiting typical clinical and pathogenic characteristics of swine mycoplasmal pneumonia in Gansu Province, China (Ho et al., 1980). *M. hyopneumoniae* strains NJ and WX which were isolated in Nanjing City and Wuxi City were also pathogenic strains verified by animal experiments. Clonal isolates of *M. hyopneumoniae* strains were cultured in KM2 cell-free liquid medium (a modified Friis medium) containing 20% (v/v) swine serum at 37°C (Liu et al., 2013). The culture was harvested by centrifugation at 12,000 rpm for 20 min at 4°C when the indicator in the medium turned yellow.

### Infection of cell lines with *M. hyopneumoniae*

STEC were seeded in 24-well plates and cultured in RPMI-1640 medium (Thermofisher Scientific, USA) supplemented with 10% (v/v) fetal bovine serum (FBS, Gibco, USA). The cells were grown at 37°C in 5% CO_2_. Confluent STEC monolayers were gently rinsed three times with sterile phosphate-buffered saline (PBS) and inoculated with *M. hyopneumoniae* strain 168. A series of 1:10 dilutions of cultures in broth with a metabolic indicator was used to estimate titers of *M. hyopneumoniae* strain 168. The dilution of the last tube to show growth was taken as the number of CCU (color change unit) (Stemke and Robertson, 1982). Total 1 × 10^8^ CCU *M. hyopneumoniae* strain 168 cells [multiplicity of infection (MOI) = 20] were washed with sterile PBS and resuspended in RPMI-1640 medium with 2% (v/v) FBS and incubated with STEC for 48 h. Supernatants were collected from each well for *M. hyopneumoniae* separation. *M. hyopneumoniae* strain 168 was cultured in RPMI-1640 medium with 2% (v/v) FBS in 24-well plates for use as the control. The assay was performed three times.

### Protein extraction

The obtained culture was centrifuged at 12,000 rpm for 20 min at 4°C. The precipitates were washed three times with sterile 10 mM Tris-HCl (pH 7.4) and resuspended in 30 μL protein extract (1.52 g thiourea [Bio-Rad], 4.2 g urea [Bio-Rad], 0.4 g CHAPS [Bio-Rad], 200 μL amphoteric electrolyte [Bio-Rad], 61.6 mg DTT [Bio-Rad], and protease inhibitor [Merck] dissolved in 10 mL ultrapure water). After vortexing for 15 s, the mixture was placed in an ice bath for 30 s, and the procedure was repeated for 15 min. The whole protein in the supernatant was obtained by centrifugation at 12,000 rpm for 30 min at 4°C. A ReadyPrep 2-D cleanup kit (Bio-Rad, USA) was used to clean up the protein samples. The protein concentration was determined using a BCA^TM^ Protein Assay Kit (Themo Scientific, USA).

### Two-dimensional electrophoresis (2-DE)

The purified proteins were quantified and redissolved with 350 μL rehydration solution (7 M urea, 2 M thiourea, 0.001% bromophenol blue; Bio-Rad), and then centrifuged (12,000 rpm for 20 min at 25°C) to remove insoluble components. The samples were loaded into a 17-cm strip (pH 3–10; Bio-Rad), and positively rehydrated at 50 V for 12 h at 20°C. Isoelectric focusing (IEF) was then carried out at 20°C as follows: slow to 250 V for 1 h; rapid to 1,000 V for 1 h; linear to 10,000 V for 3 h; rapid to 10,000 V to total 90,000 Vh; rapid to 500 V for the preservation of samples. Sodium dodecyl sulfate polyacrylamide gel electrophoresis (SDS-PAGE) was performed according to a previously described protocol (Yu et al., 2016). The 2-DE gels were repeated three times.

### Image analysis, MALDI-TOF-MS/MALDI-TOF-TOF-MS, and database search

After Coomassie blue staining, the protein spots from each gel were detected and matched automatically by the PDQuest V8.0 software followed by additional visual analysis. The intensity of individual spot for each gel was normalized relative to total valid spot intensity to eliminate gel-to-gel variation. Only spots whose abundance changed by ≥1.5-fold and a value of *p* ≤ 0.05 in Student's *t*-test in the *M. hyopneumoniae*-infected STEC compared to that non-infecting *M. hyopneumoniae* were excised from the 2-DE gel and subjected to Matrix-assisted laser desorption/ionization time-of-flight mass spectrometry (MALDI-TOF-MS/ MALDI-TOF-TOF-MS) analysis (NanJing Steed BioTechnologies Co., Ltd). Peptide mass fingerprinting data were analyzed using the MASCOT server (http://www.matrixscience.com). Peptides with a rank of 1 in the MASCOT search were considered significant and used for the combined peptide score.

### Protein-protein interaction analysis

To determine how the differential proteins contribute to virulence in *M. hyopneumoniae* and to filter the most important virulence associated factors, we summarized the putative virulence factors reported in the previously published papers (Simionatto et al., 2013; Maes et al., 2017). Then, the protein-protein interactions between the known virulence factors and the novel differential proteins identified in this study were obtained from the STRING database (http://string-db.org/newstring_cgi/show_input_page.pl), defining a “confidence score” that can be used to evaluate the confidence in the interaction. The interactions derived from textmining and experiments with a confidence score of at least 0.4 were considered for analysis. The protein-protein interaction network was visualized using Cytoscape (3.5.1).

### Recombinant differential protein and preparation of polyclonal antibody

The *ef-tu* gene encoding one of the most important novle virulence associated factors was cloned into pET-32a using homologous recombination technology (ClonExpress®IIOne Step Cloning Kit, Vazyme Biotech Co., Ltd). The sequence that overlapped with the end of the cloning site was added onto the insert through a PCR step. Table 1 lists the primers used in this study. The reconstructed plasmid was transformed into *Escherichia coli* BL21 (DE3) for isopropyl-β-d-thiogalactopyranoside (IPTG)-inducible expression, and the induced proteins were purified by Ni-chelating chromatography (GE Healthcare). Polyclonal antibody against the recombinant EF-Tu (rEF-Tu) was prepared by subcutaneously immunizing 1-month-old New Zealand white rabbits. Each rabbit was immunized a total of three times with 1 mg of purified recombinant protein emulsified in Freund's adjuvant (Sigma, USA) at 2-week intervals. Sera were collected 1 week after the third immunization. The animals used in this study met the legal and ethical requirements and were treated humanely.

**Table 1:** Primers used in this study.

**Primers name**	**Summary of Functions or Sequences (5′-3′)**
EF-Tu-F	GCCATGGCTGATATCGGATCCATGGCAGTTGTTAAAACGACA
EF-Tu-R	GTGGTGGTGGTGCTCGAGTTATTTAATAATTTCGGTAAC For recombinant expression of *ef-tu* with a restriction site of *Bam*H I and *Xho* I based on homologous recombination technology
Mhp183-F	CCAGAACCAAATTCCTTCGCTG
Mhp183-R	ACTGGCTGAACTTCATCTGGGCTA
Mhp183-P	(FAM)-AGCAGATCTTAGTCAAAGTGCCCGTG-(TAMRA)
	For real-time PCR analysis

### Western blot validation of comparative proteomics analysis

Equal amounts (40 μg for each lane) of the protein samples were separated on a 12% SDS-PAGE gel. The proteins were electrophoretically transferred onto polyvinylidene fluoride (PVDF) membranes (Bio-Rad, USA) and developed with Ponceau-S as the loading control. After washing with TBST buffer (20 Mm Tris-HCl [pH 7.6], 150 mM NaCl, and 0.1% Tween-20) and blocking with TBST buffer containing 5% skimmed milk, the membranes were incubated with primary antibody against rEF-Tu (1:2,000 dilution), followed by horseradish peroxidase (HRP)-conjugated secondary antibody (1:10,000 dilution). Signals were detected with the ECL substrate (Millipore, USA) using the ChemiDoc XRS+ system (Bio-Rad, USA). Image J software was used to calculate the optical density (OD) of the corresponding bands. The OD of EF-Tu from different samples was normalized to that of the Ponceau-S stained membrane. The level of abundance of EF-Tu in *M. hyopneumoniae* incubated with STEC is expressed as the percentage of that in *M. hyopneumoniae* cultured in KM2 medium and the three replicates were subjected to statistical analysis by SPSS 20.0.

### Detection of *M. hyopneumoniae* EF-Tu surface display using flow cytometry

Flow cytometry analysis was used to detect if EF-Tu was located on the surface of *M. hyopneumoniae* strains. In brief, three pathogenic *M. hyopneumoniae* strains, 168, NJ and WX (10^8^ CCU) were incubated with anti-rEF-Tu primary serum at a 1:100 dilution (preimmune rabbit serum was used as the negative control). The blank control was incubated with PBS instead of antibody. Then, *M. hyopneumoniae* cells were stained with fluorescein isothiocyanate (FITC)-conjugated anti-IgG. Finally, the fluorescence intensity was detected using a flow cytometer (BD Accuri® C6) (Zhu et al., 2017). The level of mean fluorescence intensity (MFI) of *M. hyopneumoniae* incubated with anti-rEF-Tu serum is expressed as the percentage of that incubated with preimmune serum. The statistical analysis was conducted using SPSS 20.0.

### Adherence of rEF-Tu to STEC

Indirect immunofluorescence assay was performed to investigate the ability of rEF-Tu to promote adherence to STEC. The cells were cultured in 24-well cell plates and fixed with cold methanol at −20°C for 20 min. Fixed cells were incubated with 100 μg of purified rEF-Tu or non-related recombinant protein HP07325, which was previously verified not to be involved in adhesion to epithelial cells (Li et al., 2017) at 37°C for 1 h. After three washes with PBS, the cells were incubated with His-tagged monoclonal antibody (Boster) at a 1:1,000 dilution. After washing three times with PBS, the cells were incubated with tetraethyl rhodamine isothiocyanate (TRITC)-tagged anti-IgG (Proteintech, 1:500 dilution) at 37°C for 30 min. Finally, the cell nuclei were stained with 6-diamidino-2-phenylindole (DAPI). Fluorescence was detected using a fluorescence microscope (Zeiss, Germany). The assay was performed in triplicate.

### Adherence inhibition assay of antibody against rEF-Tu

*M. hyopneumoniae* cells (1 × 10^7^ CCU/mL) were washed three times with PBS and pre-incubated with the polyclonal antibody (1:20 dilution) against rEF-Tu at 37°C for 30 min. *M. hyopneumoniae* that had been pre-incubated with preimmune sera (1:20 dilution) were used as the control. The bacterial suspension in RPMI-1640 medium was added to each well of 24-well cell plates with confluent STEC, and the plates were centrifuged at 800 × g for 10 min and incubated at 4°C for 2 h. Following incubation, the wells were washed three times with PBS to remove unbound *M. hyopneumoniae* cells. Then the cells in the wells were treated with lysis buffer containing 0.1% trypsin and 0.025% (v/v) Triton X-100, followed by bacterial genome extraction and real-time PCR for bacteria counting. Real-time PCR was performed using a QuantStudio® 5 Real-Time PCR System according to a previous method, with a slight modification (Wu et al., 2012). Table 1 lists the primers used. The assay was performed in triplicate, and the data were analyzed using Student's *t*-test using SPSS 20.0. For all tests, a value of *p* ≤ 0.05 was considered statistically significant.

### Analysis of rEF-Tu binding to fibronectin

To explore if *M. hyopneumoniae* EF-Tu could bind to fibronectin, the protein-protein interactions method far-Western blot (far-WB) was performed. Total 20 μg recombinant proteins, including rEF-Tu and a negative control MRP-D2, which was previously verified not to bind to fibronectin (Li et al., 2017) were separated by SDS-PAGE and transferred to a polyvinylidene fluoride (PVDF) membrane (Li et al., 2015). After blocked with 5% (w/v) skimmed milk, the membrane was incubated with 5 μg/mL fibroenctin (Sigma), followed by incubation with rabbit anti-fibronectin antibody (Boster; 1 μg/mL) as the primary antibody, and horseradish peroxidase (HRP)-conjugated goat anti-rabbit IgG (Boster; 1:5,000 dilution) as the secondary antibody. Finally, the membrane was developed with ECL substrate using a ChemiDoc XRS+ system. Polyclonal antibody against rEF-Tu was used as the positive control with the same process.

### Surface plasmon resonance analysis

The rEF-Tu and fibronectin interaction dynamics was further investigated in real time by surface plasmon resonance (SPR) experiment on a Biacore™ X100 Plus instrument (GE Healthcare). Fibronectin was diluted to 10 μg/mL in 10 mM sodium acetate (pH 4.0) and covalently linked to the carboxylmethylated dextran matrix of a sensor chip CM5 as ligands using an amine coupling kit (Biacore AB). Immobilization of soluble fibronectin generated resonance units (RU) of 2868. The binding kinetics was measured with the increasing concentrations (0–200 μg/mL) of the analyte (rEF-Tu) in running buffer (HBS-EP + [10 mM HEPES, 150 mM NaCl, 3 mM EDTA, 0.05% (v/v) surfactant P20]; Biacore AB) at a flow rate of 30 μL/min for 180 s over immobilized fibronectin at 20°C. The dissociation phase was monitored for 1,000 s by allowing buffer to flow over the chip. Association kinetics were analyzed manually using Biacore™ X100 Control Software (Deutscher et al., 2010).

### Immunohistochemistry

Fibronectin is not only an abundant glycoprotein deposited on cell surfaces (McDonald, 1988), but also an important component involved in wound repair of respiratory epithelial cells (Coraux et al., 2008). Therefore, if the pigs are infected with *M. hyopneumoniae* which would damage cilia of the respiratory tract, there may be more fibronectin around the respiratory epithelial cells. Then the abundant fibronectin can be utilized by the bacteria for adhesion. To investigate if there are some differences in fibronectin abundance in bronchioles of infected and uninfected pigs and to explore the predominant distribution of fibronectin in bronchioles, the immunohistochemistry was performed. Polyformaldehyde-fixed bronchial tissues were placed into histology cassettes and embedded in paraffin. Then, 4-mm-thick sections were cut for bronchial transection and processed for immunohistochemical staining. To stain the sections for fibronectin, the slides were blocked with 5% BSA and incubated with a 1:100 dilution of anti-fibronectin antibody at 4°C in a humidified chamber. Following each incubation step, the slides were washed three times with sterile PBS-Tween. The negative control slides were treated identically except that PBS was used instead of the primary antibody. Bound antibody was detected with a chromagen solution containing 3-amino-9-ethylcarbazole and 0.015% hydrogen peroxide in dimethylformamide. The slides were counterstained with haematoxylin (Seymour et al., 2012).

### Adherence inhibition assay of antibody against fibronectin

The confluent STEC in 24-well cell plates was washed three times with PBS and pre-incubated with the antibody against fibronectin (1:20 dilution) at 37°C for 30 min. The RPMI-1640 medium was used instead of fibronectin antibody as the negative control. *M. hyopneumoniae* cells (1 × 10^7^ CCU/mL) were washed three times with PBS. The bacteria resuspended in RPMI-1640 medium was added to the 24-well cell plates and incubated at 4°C for 2 h. After incubation, the wells were washed three times with PBS to remove unbound *M. hyopneumoniae* cells. Then the cells in the wells were treated with lysis buffer containing 0.1% trypsin and 0.025% (v/v) Triton X-100. The following bacterial genome extraction and real-time PCR for bacteria counting were conducted according to the method in section Adherence Inhibition Assay of Antibody Against rEF-Tu. The assay was performed in triplicate, and the data were analyzed using Student's *t*-test using SPSS 20.0. A value of *p* ≤ 0.05 was considered statistically significant.

## Results

### Comparative proteomics analysis found six differential abundance proteins

**Figure 1:**
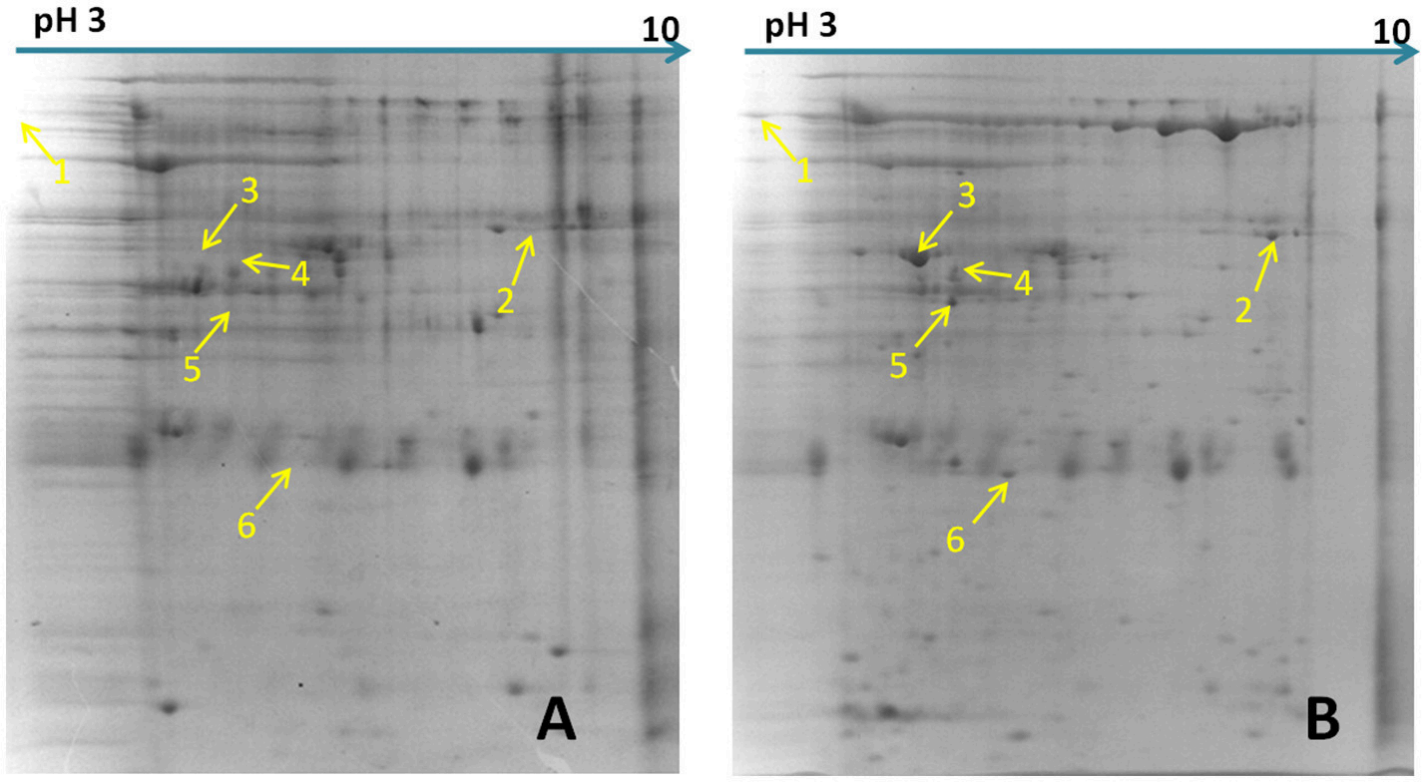
Differential abundance proteins analyzed by two-dimensional electrophoresis (2-DE). **(A)** Bacterial proteins of *M. hyopneumoniae* strain 168 cultured in KM2 medium. **(B)** Bacterial proteins of *M. hyopneumoniae* strain 168 incubated with swine tracheal epithelial cells (STEC). Yellow arrows on the gel images indicate the six protein spots that show increased abundance (proteins whose abundance changed by ≥1.5-fold) after infection of STEC with *M. hyopneumoniae*.

The differential proteins that showed increased abundance (proteins whose abundance changed by ≥1.5-fold) in the *M. hyopneumoniae*-infected STEC were subjected to MALDI-TOF-MS/MALDI-TOF-TOF-MS analysis. Six novel differential abundance proteins were successfully identified. They were YX2, pyruvate dehydrogenase E1-alpha subunit, EF-Tu, hypothesis protein MHJ-0662, enolase, and adenine phosphoribosyltransferase. Among them, EF-Tu showed a relatively higher expression in *M. hyopneumoniae*-infected STEC in 2D gels (Figure 1) and obtained a higher score in mass spectrometry analysis (Table 2).

**Table 2:** The proteins with significant changes in abundance.

**Spot No**.	**Protein description**	**Accession**	**Score**	**Mass**	**pI**
1	YX2	AF279292	116	63766	8.15
2	Hypothesis protein MHJ-0662	AAZ44745	169	135249	6.73
3	Elongation factor thermo unstable	WP_011284269	201	43633	6.30
4	Enolase	ADQ90480	157	49359	6.00
5	Pyruvate dehydrogenase E1-alpha subunit	WP_011206102	106	42423	5.24
6	Adenine phosphoribosyltransferase	WP_011206103	70	18640	8.67

### Network analysis of the novel differential abundance proteins and known putative virulence factors

**Figure 2:**
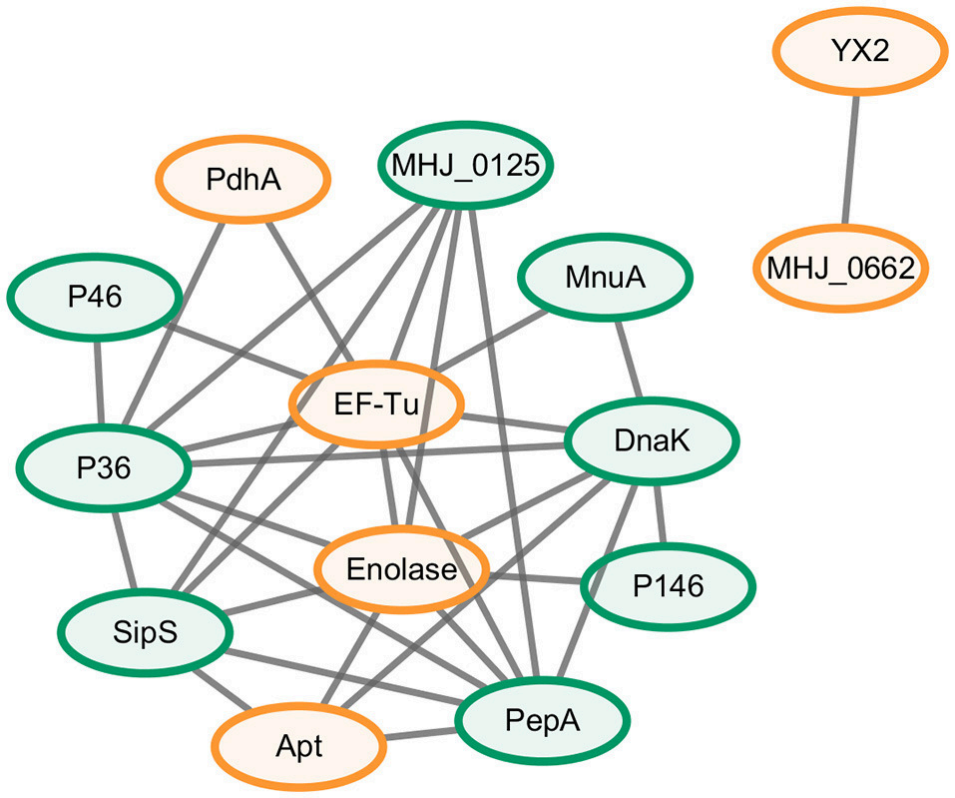
Interaction networks of the novel differential abundance proteins and known putative virulence factors. Protein-protein interactions derived from String database of sources of textmining and experiments with a confidence score ≥0.4 were displayed. Green nodes represent the known putative virulence factors collected from published papers and orange nodes represent the novel differential proteins with increased abundance when *M. hyopneumoniae* strain 168 cells were incubated with STEC. Gray lines represent interactions between two nodes.

A protein-protein interaction network was constructed to explore the relationship between the novel differential abundance proteins identified in this study and *M. hyopneumoniae* virulence and to filter the most important virulence associated factors. The results revealed a total of 32 direct physical interactions among the 14 nodes (Figure 2 and Data Sheet 1).These results indicated that all the six proteins with increased abundance in the infected cells were implicated in the interaction network. Four of them even established connection with the known virulence factors. Thereinto, EF-Tu showed the most links to other virulence factors. It connected previously known putative virulence factors and the novel differential abundance proteins and formed important hub proteins in the network. The results indicated that these six proteins with differential abundance were involved in *M. hyopneumoniae* virulence, and EF-Tu was one of the most important proteins in the virulence network.

### Western blot validation of comparative proteomics analysis

**Figure 3:**
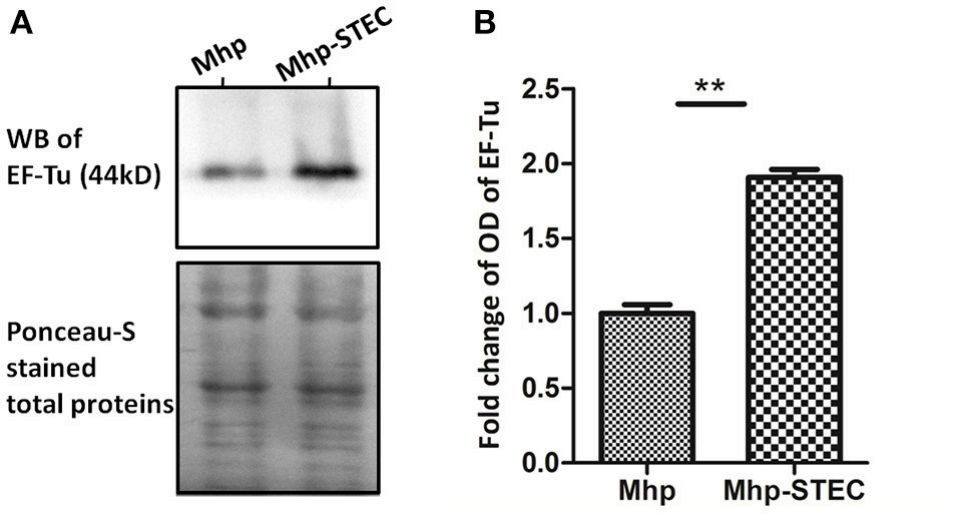
Western blot analysis of the proteomics data. **(A)** Equal amounts (40 μg for each lane) of bacterial proteins from each sample were separated on a 12% SDS-PAGE gel and subjected to Ponceau-S stainning and western blot analysis. The left lane was loaded with the bacterial proteins of *M. hyopneumoniae* cultured in KM2 medium. The right lane was loaded with bacterial proteins of *M. hyopneumoniae* incubated with STEC. The differential abundance protein EF-Tu (44 kDa) was analyzed using its polyclonal antibody. The Ponceau-S stained membrane was used as the loading control. Protein bands were visualized using ECL substrate. **(B)** Image J software was used to calculate the optical density of the corresponding bands in the blots. The optical density of the corresponding bands was normalized to the total proteins of Ponceau-S staining of the same membrane. The level of abundance of EF-Tu in *M. hyopneumoniae* incubated with STEC is expressed as the percentage of that in *M. hyopneumoniae* cultured in KM2 medium. The asterisk above the charts stands for statistically significant differences.

EF-Tu was selected for validation of the comparative proteomics analysis and further studies considering that it showed a relatively higher expression in *M. hyopneumoniae*-infected STEC, a higher score on mass spectrometry analysis and a stronger connection to the known virulence factors in the STRING network. The results of the western blot showed that the OD of EF-Tu normalized by Ponceau-S stained total proteins was increased significantly when *M. hyopneumoniae* interacted with STEC (Figure 3). Thus, it showed consistency of the upregulation trend of EF-Tu between the two approaches. It confirmed the increased abundance of EF-Tu in *M. hyopneumoniae*-infected STEC and supported the results of the proteomics analysis to some extent.

### Flow cytometry analysis detected the surface location of EF-Tu

**Figure 4:**
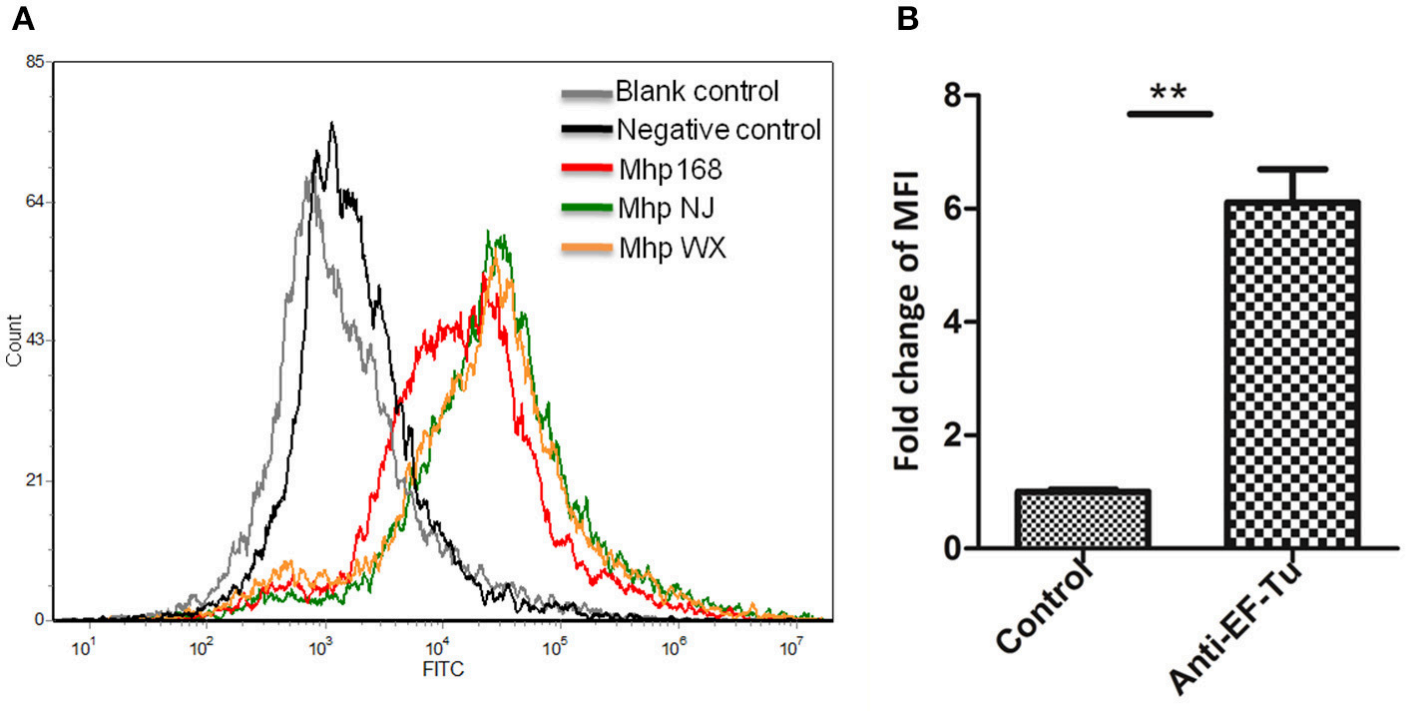
Detection of *M. hyopneumoniae* EF-Tu surface display using flow cytometry. **(A)** Blank control, bacteria treated with PBS alone; negative control, bacteria treated with preimmune serum; Mhp strain 168, NJ, and WX: bacteria treated with anti-rEF-Tu serum. **(B)** The level of mean fluorescence intensity (MFI) of *M. hyopneumoniae* incubated with anti-rEF-Tu sera is expressed as the percentage of that incubated with preimmune sera. The asterisk above the charts stands for statistically significant differences.

Significant fluorescence was detected in all three pathogenic *M. hyopneumoniae* strains incubated with anti-rEF-Tu serum. The fluorescence intensity of *M. hyopneumoniae* strain treated with preimmune serum was close to that of unlabeled *M. hyopneumoniae* strain, whereas the MFI of bacteria treated with anti- rEF-Tu serum was more than 6 times that of bacterial cells treated with preimmune serum (Figure 4). The significant differences in fluorescence intensity indicated that EF-Tu was present on the bacterial cell surface of all the three pathogenic *M. hyopneumoniae* strains examined here.

### Adherence of rEF-Tu to STEC detected by indirect immunofluorescence

**Figure 5:**
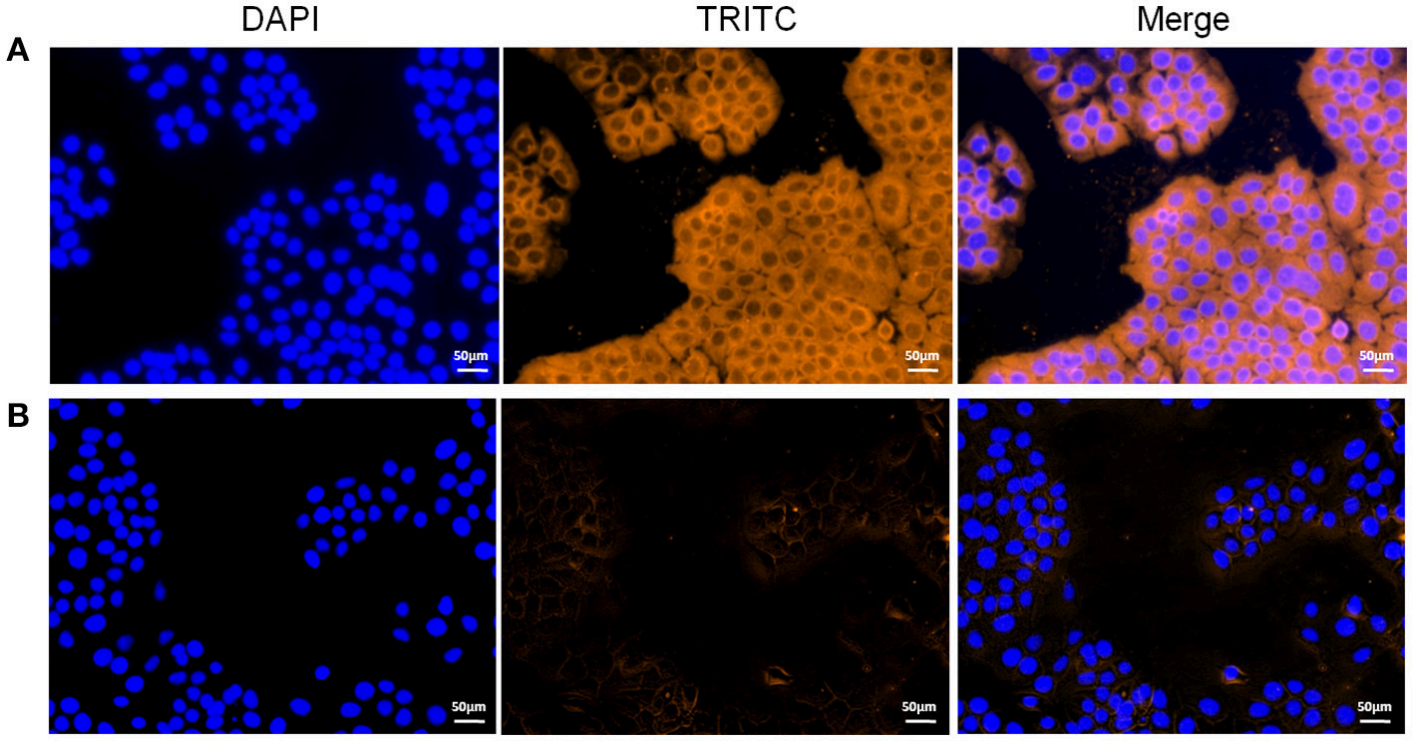
Role of rEF-Tu in adhesion of *M. hyopneumoniae* to STEC. **(A,B)** Blue color indicates the STEC nucleus, orange indicates **(A)** rEF-Tu adhering to STEC membranes, **(B)** non-related recombinant protein HP07325 (negative control) adhering to STEC membranes.

To explore the potential mechanism(s) by which the EF-Tu surface protein affected virulence, we used indirect immunofluorescence to determine whether or not rEF-Tu could adhere to STEC. Significant fluorescence was detected on the cell surface of STEC incubated with rEF-Tu (Figure 5A); meanwhile, no specific fluorescence was observed around the DAPI-stained cell nuclei in the negative control, non-related recombinant protein HP07325 (Figure 5B). The results provided evidence that rEF-Tu could specifically bind to STEC cell membranes.

### Anti-EF-Tu antibody inhibition assay

**Figure 6:**
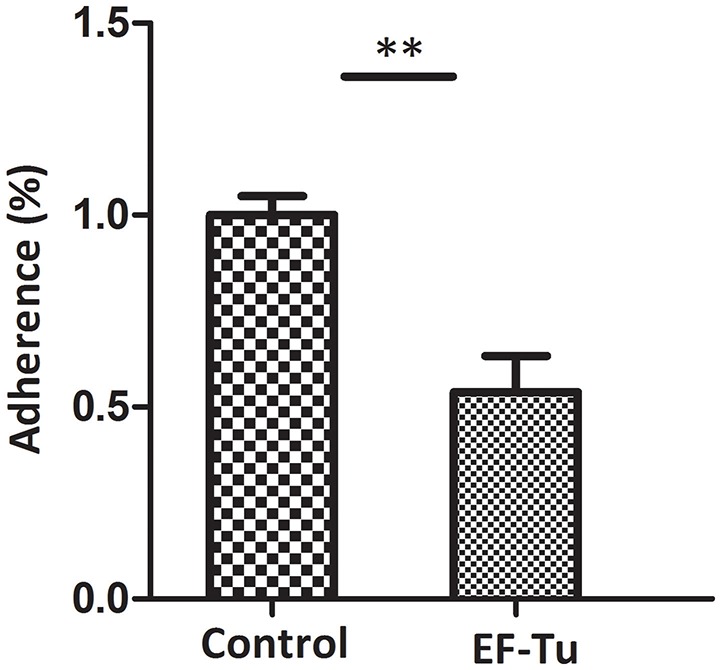
Adhesion inhibition assay of rEF-Tu antibody. Adhesion rate: number of bacteria recovered in the cells incubated with anti-rEF-Tu sera/number of bacteria recovered in the group incubated with preimmune sera × 100%. Data are expressed as means ± SD of at least three experiments with samples performed in triplicate. The asterisk stands for statistically significant differences.

The antibody inhibition assay was used to further assess the contribution of EF-Tu to the adhesion of *M. hyopneumoniae*. Polyclonal antibody against rEF-Tu and preimmune sera were used to incubate with *M. hyopneumoniae* cells respectively before adherence to STEC. The level of adherence of *M. hyopneumoniae* incubated with anti-rEF-Tu sera is expressed as the percentage of *M. hyopneumoniae* adherence with preimmune sera. The results revealed that after incubation with anti-rEF-Tu antibody, the adherence efficiency of *M. hyopneumoniae* to STEC showed a 46% (*P* < 0.05) reduction compared to that incubated with preimmune sera (Figure 6). These results reconfirm that EF-Tu plays an indispensable role in the adherence of *M. hyopneumoniae* to host cells.

### rEF-Tu showed fibronectin-binding activities

**Figure 7:**
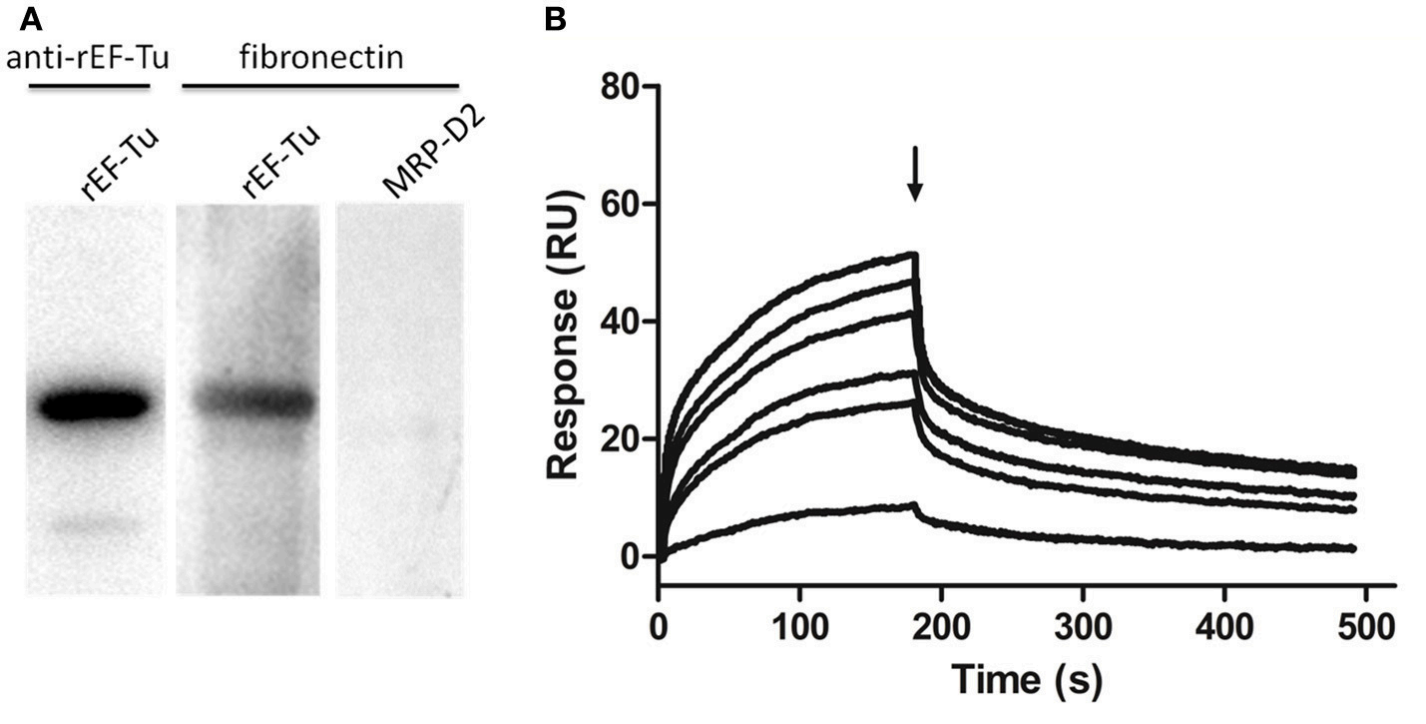
rEF-Tu and fibronectin interaction analysis by Far-WB and SPR analysis. **(A)** Far-WB analysis of rEF-Tu with fibronectin. The first lane: PVDF membrane with transferred rEF-Tu protein incubated with anti- rEF-Tu antibody as a positive control; the second lane: PVDF membrane with transferred rEF-Tu protein incubated with fibronectin and anti-fibronectin antibody; the third lane: PVDF membrane with transferred a non-related recombinant protein MRP-D2 (negative control) incubated with fibronectin and anti-fibronectin antibody. Protein bands were visualized using ECL substrate. **(B)** Binding activity of rEF-Tu to fibronectin by SPR analysis. Sensorgrams depict binding of immobilized fibronectin by rEF-Tu. Increasing concentrations of the rEF-Tu was injected at 10, 25, 50, 100, 150, and 200 μg/mL at a flow rate of 30 μL/min for 180 s over immobilized fibronectin. Arrows indicate the end of the injection period at which point dissociation of rEF-Tu from fibronectin can be observed. The reaction is shown in resonance units (RU).

To determine the STEC components that Interact with EF-Tu, we examined the fibronectin-binding activities of rEF-Tu with far-WB analysis. The corresponding bands were observed in both reactions of rEF-Tu to anti-EF-Tu antibody (positive control) and to fibronectin, while no specific reaction was observed in the negative control MRP-D2, a non-related recombinant protein which was verified not to bind to fibronectin in a previous study (Li et al., 2017). The analysis indicated that rEF-Tu could specifically bind to fibronectin (Figure 7A). The SPR analysis was then used to observe real-time interactions between rEF-Tu and fibronectin (Figure 7B). The specific dose-dependent binding component that describes binding of rEF-Tu to immobilized fibronectin is characterized by a second-order rate constant *k*_a_ = 3470 ± 580 M^−1^·s^−1^ and a *K*_D_ = 605 ± 35 nM. Both of these values are consistent with expectations for a specific, moderately strong interaction between proteins of this size (Deutscher et al., 2010). rEF-Tu binds to fibronectin in a dose-dependent and physiologically relevant manner (Figure 7B).

### Abundant fibronectin is present in the ciliary borders of bronchioles

**Figure 8:**
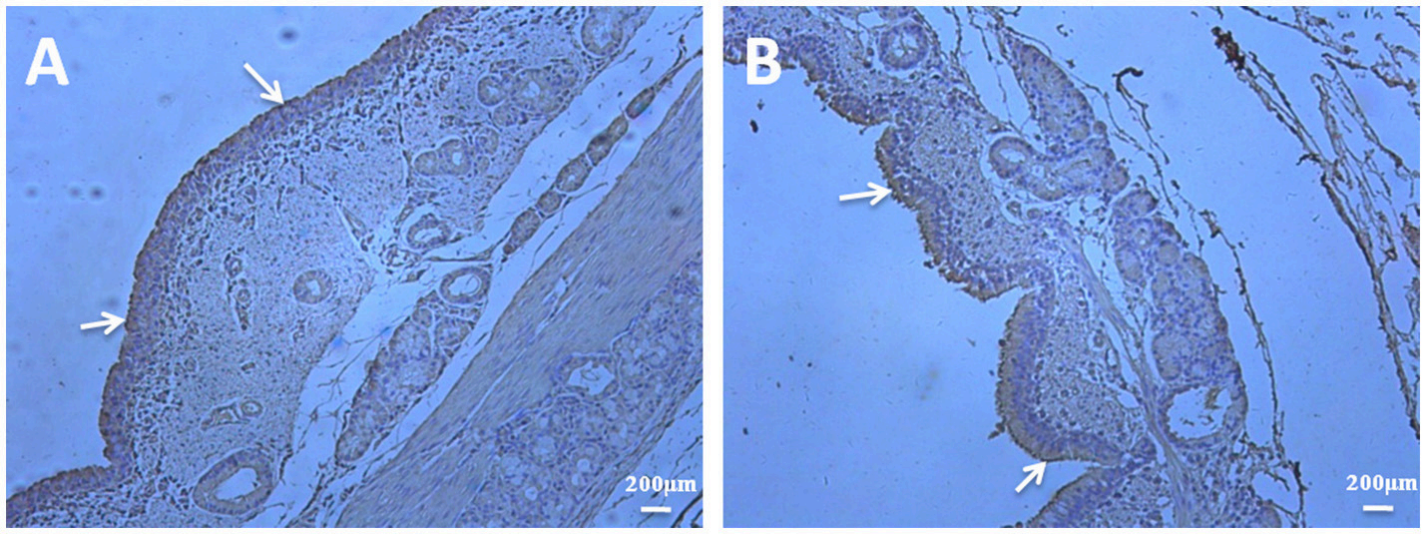
Fibronectin is predominantly present in the ciliary borders of porcine bronchioles. Immunohistochemical staining of porcine bronchioles sections from unchallenged **(A)** and *M. hyopneumoniae*-challenged **(B)** pigs was performed with anti-fibronectin antibody. The presence of brown stain highlights the location of fibronectin. A widespread distribution of fibronectin can be observed in the bronchioles sections of all samples, especially the ciliary borders of the bronchioles (as indicated by the white arrowheads).

To investigate if there are some differences in abundance of fibronectin in bronchioles when infected by *M. hyopneumoniae* and to explore the predominant distribution of fibronectin, the immunohistochemical staining was performed. Bronchioles sections obtained from both unchallenged and *M. hyopneumoniae*-challenged pigs were subjected to immunohistochemical staining. Sections were initially stained with an *M. hyopneumoniae*-specific antibody to confirm the presence and absence of infection in challenged and unchallenged pigs, respectively. All samples, from both infected and uninfected animals, stained positive for fibronectin. Fibronectin levels in challenged and unchallenged pigs did not appear to vary. Fibronectin is available at the colonization site along the airways, especially the ciliary borders, which is a prefered place for *M. hyopneumoniae* colonization (Figure 8).

### Block of fibronectin decreased the binding of *M. hyopneumoniae* to STEC

**Figure 9:**
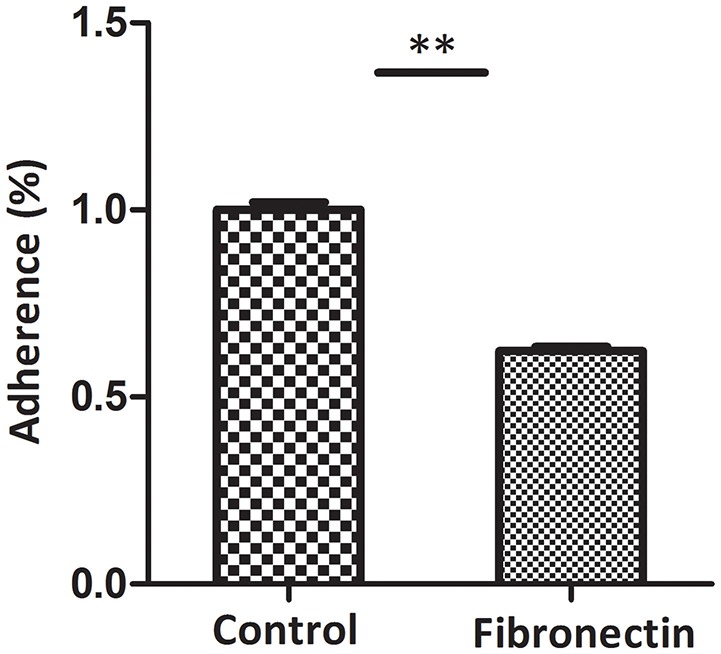
Block of fibronectin decreased the binding of *M. hyopneumoniae* to STEC. Adhesion rate: The number of bacteria recovered in the cells incubated with anti-fibronectin antibody/number of bacteria recovered in the cells incubated with RPMI-1640 medium × 100%. Data are expressed as means ± SD of at least three experiments with samples performed in triplicate. The asterisk stands for statistically significant differences.

Fibronectin antibody inhibition assay was used to assess the contribution of fibronectin to the adhesion of *M. hyopneumoniae* to STEC. The level of adherence of *M. hyopneumoniae* to STEC incubated with anti-fibronectin antibody is expressed as the percentage of that without antibody. The results revealed that after incubation with antibody against fibronectin in STEC, the binding of *M. hyopneumoniae* to cell surface was decreased significantly. The adherence efficiency of *M. hyopneumoniae* to STEC showed a 38% (*P* < 0.05) reduction compared to that incubated without antibody (Figure 9). Fibronectin was verified as one of the host cell receptors for *M. hyopneumoniae* adhesion.

## Discussion

Over the past several decades, although some putative virulence factors have been associated with *M. hyopneumoniae* adhesion, the pathogenesis of this bacterium remains unclear.

In this study, six proteins with significantly increased abundance were identified from *M. hyopneumoniae* strain 168 when infecting STEC. Among them, EF-Tu showed a relatively higher expression in *M. hyopneumoniae*-infected STEC and obtained a higher score in mass spectrometry analysis. A virulence-associated network was thus established using these six proteins and the reported putative virulence factors collected from previously published papers. One of the hubs involved in *M. hyopneumoniae* virulence which showed the most links to the other virulence associated factors in this network, EF-Tu was identified. Furthermore, analysis of *M. hyopneumoniae* cells cultured *in vitro* indicated that EF-Tu was accessible on the *M. hyopneumoniae* surface and that EF-Tu is indispensable for adherence of *M. hyopneumoniae* to STEC. rEF-Tu was found to bind to fibronectin with a specific and moderately strong interaction. Fibronectin which is available in respiratory tract, could be one of the receptors for EF-Tu.

In general, a pathogenic microorganism initiates infection by adhering to the host target, and involves complex pathogen-host interactions and molecular cross-talk between microbial adhesins and host cell receptors (Finlay and Cossart, 1997). As the adherence to host tissues is an important prerequisite for colonization and subsequent disease development by pathogenic bacteria, adhesins are of crucial importance for *M. hyopneumoniae* infection (Razin and Jacobs, 1992).

EF-Tu is the most abundant bacterial protein (Jacobson and Rosenbusch, 1976), constituting approximately 10% of *M. pneumoniae* total protein content (Regula et al., 2000). The canonical function of the conserved cytoplasmic protein is acting as a GTPase on the binding of aminoacyl-tRNA to the ribosome-mRNA complex during translation elongation in protein synthesis (Voorhees and Ramakrishnan, 2013). In addition to its well-defined enzymatic activities in protein synthesis, EF-Tu also moonlights on the cell surface as an important adhesin in numerous prokaryotes. For example, 17% of the total EF-Tu in *M. pneumoniae* has been detected in the membrane fraction (Dallo et al., 2002). EF-Tu is also identified as a major cell surface protein in *Mycobacterium leprae* (Marques et al., 1998). The ability of EF-Tu to function as a fibronectin-binding protein in several bacterial pathogens, including *M. pneumoniae* (Balasubramanian et al., 2008), *Streptococcus suis* (Li et al., 2015), and *Mycobacterium avium* (Viale et al., 2014), provided evidence of its biological versatility. A previous study and our flow cytometry analysis confirmed the membrane distribution of EF-Tu in *M. hyopneumoniae* (Reolon et al., 2014). Adherence of viable *M. hyopneumoniae* to STEC was inhibited by anti-rEF-Tu antisera. Another comparative proteomics analysis also showed the association of abundance of EF-Tu to *M. hyopneumoniae* adhesion. Seven proteins including EF-Tu showed increased abundance in virulent *M. hyopneumoniae* strain 168 which showed stronger adhesion to STEC compared with the less invasive strain 168L which is obtained from continuous passage of strain 168 (unpublished data). These results demonstrate that *M. hyopneumoniae* may increase abundance of EF-Tu to help better attach to the host cells to some extent. Thus, *M. hyopneumoniae* EF-Tu, in addition to its major cytoplasmic, biosynthetic, and metabolic roles, can translocate to the surface and moonlight as an important adhesion factor.

Bacterial adherence is frequently mediated by a spectrum of host targets, often the extracellular matrix (ECM). Interaction with the ECM components, with the most common being fibronectin, may influence the initiation, facilitate colonization, and establishment of infection, and express important virulence factors (Patti and Höök, 1994; Schwarz-Linek et al., 2004). Fibronectin is a glycoprotein that often exists as a soluble dimer in body fluids, or as an insoluble multimer in the ECM. The capacity to bind to fibronectin is widespread in bacterial pathogens. Among mycoplasmas, the first fibronectin interaction was identified in *M. penetrans* (Girón et al., 1996), followed by *M. pneumoniae* (Dallo et al., 2002) and *M. hyopneumoniae* (Deutscher et al., 2010). Many fibronectin-binding proteins have been identified, such as the microbial surface components recognizing adhesive matrix molecules (MSCRAMM) family of proteins identified in *Staphylococcus* (Grundmeier et al., 2004) and *Streptococcus* (Lindgren et al., 1992). The use of the mammalian fibronectin system by pathogens has become a widely accepted virulence strategy. The capacity of *M. hyopneumoniae* to bind to fibronectin therefore warrants further investigation.

Using far-WB analysis, rEF-Tu was found to be able to bind to fibronectin. The dissociation constant of rEF-Tu determined by SPR displayed values with a specific and moderately strong interaction. Furthermore, block of fibronectin in STEC decreased *M. hyopneumoniae* adherence to cell surface significantly. Immunohistochemical staining of bronchioles showed that fibronectin is more abundant in the ciliary borders of the bronchioles, which is a prefered place for *M. hyopneumoniae* colonization. Thus, fibronectin was verified one of the host cell receptors for *M. hyopneumoniae* adhesion.

In summary, using system-wide methodologies, EF-Tu was found to act as an important hub in the virulence-associated network of *M. hyopneumoniae*. Further analysis revealed that *M. hyopneumoniae* displays EF-Tu on the surface to bind to host fibronectin with a moderately high affinity, thereby promoting adherence to STEC. This binding was markedly reduced by pretreatment of bacterial cells with anti-rEF-Tu sera or pretreatment of STEC with anti-fibronectin antibody. The binding between EF-Tu with fibronectin was proven to contribute to the adhesion of *M. hyopneumoniae* to STEC.

However, in the competitive adhesion inhibition assay, only partial inhibition of EF-Tu was observed. Since several other proteins, such as P97 (Zhang et al., 1995), P102 (Adams et al., 2005), Mhp683 (Bogema et al., 2011), and so on have also been verified to contribute to the adhesion process of *M. hyopneumoniae*, it can be speculated that when EF-Tu is blocked, the adhesion of *M. hyopneumoniae* still partly exists.

In conclusion, moonlighting proteins located in various parts of the cell, not only in the cytoplasm but also on the cell membrane, have been suggested to be associated with *M. hyopneumoniae* virulence. A lot remains to be understood about how proteins, especially those lacking signal motifs, localize on bacterial cell surfaces. *M. hyopneumoniae* moonlighting proteins and their roles in infection and immunity should be further studied in the future.

## Author contributions

YY completes the study of the pathogenic mechanism of EF-Tu and prepares the manuscript. The comparative proteomics analysis is done by HW. JW, and BL perform the cell adhesion experiments. MW prepares the polyclonal antibody. ZF, JX, and GS modify the manuscript. QX and ML supervise and guide this work.

### Conflict of interest statement

The authors declare that the research was conducted in the absence of any commercial or financial relationships that could be construed as a potential conflict of interest.

## References

[B1] Adams C. Pitzer J. Minion F. C. (2005). *In vivo* expression analysis of the P97 and P102 paralog families of *Mycoplasma hyopneumoniae*. Infect. Immun. 73, 7784–7787. 10.1128/IAI.73.11.7784-7787.2005 16239586 PMC1273896

[B2] Balasubramanian S. Kannan T. R. Baseman J. B. (2008). The surface-exposed carboxyl region of *Mycoplasma pneumoniae* elongation factor Tu interacts with fibronectin. Infect. Immun. 76, 3116–3123. 10.1128/IAI.00173-08 18411296 PMC2446705

[B3] Bogema D. R. Scott N. E. Padula M. P. Tacchi J. L. Raymond B. B. Jenkins C.. (2011). Sequence TTKF downward arrow QE defines the site of proteolytic cleavage in Mhp683 protein, a novel glycosaminoglycan and cilium adhesin of *Mycoplasma hyopneumoniae*. J. Biol. Chem. 286, 41217–41229. 10.1074/jbc.M111.226084 21969369 PMC3308835

[B4] Burnett T. A. Dinkla K. Rohde M. Chhatwal G. S. Uphoff C. Srivastava M.. (2006). P159 is a proteolytically processed, surface adhesin of *Mycoplasma hyopneumoniae*: defined domains of P159 bind heparin and promote adherence to eukaryote cells. Mol. Microbiol. 60, 669–686. 10.1111/j.1365-2958.2006.05139.x 16629669

[B5] Coraux C. Roux J. Jolly T. Birembaut P. (2008). Epithelial cell-extracellular matrix interactions and stem cells in airway epithelial regeneration. Proc. Am. Thorac. Soc. 5, 689–694. 10.1513/pats.200801-010AW 18684718

[B6] Dallo S. F. Kannan T. R. Blaylock M. W. Baseman J. B. (2002). Elongation factor Tu and E1 beta subunit of pyruvate dehydrogenase complex act as fibronectin binding proteins in *Mycoplasma pneumoniae*. Mol. Microbiol. 46, 1041–1051. 10.1046/j.1365-2958.2002.03207.x 12421310

[B7] DeBey M. C. Ross R. F. (1994). Ciliostasis and loss of cilia induced by *Mycoplasma hyopneumoniae* in porcine tracheal organ cultures. Infect. Immun. 62, 5312–5318. 7960110 10.1128/iai.62.12.5312-5318.1994 PMC303270

[B8] Deutscher A. T. Jenkins C. Minion F. C. Seymour L. M. Padula M. P. Dixon N. E.. (2010). Repeat regions R1 and R2 in the P97 paralogue Mhp271 of *Mycoplasma hyopneumoniae* bind heparin, fibronectin and porcine cilia. Mol. Microbiol. 78, 444–458. 10.1111/j.1365-2958.2010.07345.x 20879998

[B9] Deutscher A. T. Tacchi J. L. Minion F. C. Padula M. P. Crossett B. Bogema D. R.. (2012). *Mycoplasma hyopneumoniae* surface proteins Mhp385 and Mhp384 bind host cilia and glycosaminoglycans and are endoproteolytically processed by proteases that recognize different cleavage motifs. J. Proteome Res. 11, 1924–1936. 10.1021/pr201115v 22229926

[B10] Finlay B. B. Cossart P. (1997). Exploitation of mammalian host cell functions by bacterial pathogens. Science 276, 718–725. 9115192 10.1126/science.276.5313.718

[B11] Girón J. A. Lange M. Baseman J. B. (1996). Adherence, fibronectin binding, and induction of cytoskeleton reorganization in cultured human cells by Mycoplasma penetrans. Infect. Immun. 64, 197–208. 8557340 10.1128/iai.64.1.197-208.1996 PMC173746

[B12] Grundmeier M. Hussain M. Becker P. Heilmann C. Peters G. Sinha B. (2004). Truncation of fibronectin-binding proteins in Staphylococcus aureus strain Newman leads to deficient adherence and host cell invasion due to loss of the cell wall anchor function. Infect. Immun. 72, 7155–7163. 10.1128/IAI.72.12.7155-7163.2004 15557640 PMC529102

[B13] Ho C. Chu T. Chin H. Mao H. Yeh A. Chen C. (1980). Microagglutination test for the diagnosis of swine mycoplasmal pneumonia and the identification of mycoplasmas. Acta Veterin. Zootech. Sin. 11, 175–186.

[B14] Jacobson G. R. Rosenbusch J. P. (1976). Abundance and membrane association of elongation factor Tu in *E*. coli. Nature 261, 23–26. 775340 10.1038/261023a0

[B15] Li Q. Fu Y. Ma C. He Y. Yu Y. Du D.. (2017). The non-conserved region of MRP is involved in the virulence of Streptococcus suis serotype 2. Virulence 8, 1274–1289. 10.1080/21505594.2017.1313373 28362221 PMC5711419

[B16] Li Q. Liu H. Du D. Yu Y. Ma C. Jiao F.. (2015). Identification of Novel Laminin- and Fibronectin-binding Proteins by Far-Western Blot: Capturing the Adhesins of Streptococcus suis Type 2. Front. Cell. Infect. Microbiol. 5:82. 10.3389/fcimb.2015.00082 26636044 PMC4644805

[B17] Lindgren P. E. Speziale P. McGavin M. Monstein H. J. Höök M. Visai L.. (1992). Cloning and expression of two different genes from Streptococcus dysgalactiae encoding fibronectin receptors. J. Biol. Chem. 267, 1924–1931. 1530943

[B18] Liu W. Xiao S. Li M. Guo S. Li S. Luo R.. (2013). Comparative genomic analyses of *Mycoplasma hyopneumoniae* pathogenic 168 strain and its high-passaged attenuated strain. BMC Genomics 14:80. 10.1186/1471-2164-14-80 23384176 PMC3626624

[B19] Maes D. Sibila M. Kuhnert P. Segalés J. Haesebrouck F. Pieters M. (2017). Update on mycoplasma hyopneumoniae infections in pigs: knowledge gaps for improved disease control. Transbound. Emerg. Dis. [Epub ahead of print]. 10.1111/tbed.12677 28834294

[B20] Marques M. A. Chitale S. Brennan P. J. Pessolani M. C. (1998). Mapping and identification of the major cell wall-associated components of *Mycobacterium leprae*. Infect. Immun. 66, 2625–2631. 9596726 10.1128/iai.66.6.2625-2631.1998 PMC108248

[B21] Mayor D. Zeeh F. Frey J. Kuhnert P. (2007). Diversity of *Mycoplasma hyopneumoniae* in pig farms revealed by direct molecular typing of clinical material. Vet. Res. 38, 391–398. 10.1051/vetres:2007006 17506969

[B22] McDonald J. A. (1988). Extracellular matrix assembly. Annu. Rev. Cell Biol. 4, 183–207. 2848551 10.1146/annurev.cb.04.110188.001151

[B23] Patti J. M. Höök M. (1994). Microbial adhesins recognizing extracellular matrix macromolecules. Curr. Opin. Cell Biol. 6, 752–758. 10.1016/0955-0674(94)90104-X 7833055

[B24] Razin S. Jacobs E. (1992). Mycoplasma adhesion. J. Gen. Microbiol. 138, 407–422. 10.1099/00221287-138-3-407 1593256

[B25] Regula J. T. Ueberle B. Boguth G. Görg A. Schnölzer M. Herrmann R.. (2000). Towards a two-dimensional proteome map of *Mycoplasma pneumoniae*. Electrophoresis 21, 3765–3780. 10.1002/1522-2683(200011)21:17<3765::AID-ELPS3765>3.0.CO;2-6 11271496

[B26] Reolon L. A. Martello C. L. Schrank I. S. Ferreira H. B.. (2014). Survey of surface proteins from the pathogenic *Mycoplasma hyopneumoniae* strain 7448 using a biotin cell surface labeling approach. PLoS ONE 9:e112596. 10.1371/journal.pone.0112596 25386928 PMC4227723

[B27] Schwarz-Linek U. Höök M. Potts J. R. (2004). The molecular basis of fibronectin-mediated bacterial adherence to host cells. Mol. Microbiol. 52, 631–641. 10.1111/j.1365-2958.2004.04027.x 15101971

[B28] Seymour L. M. Falconer L. Deutscher A. T. Minion F. C. Padula M. P. Dixon N. E.. (2011). Mhp107 is a member of the multifunctional adhesin family of *Mycoplasma hyopneumoniae*. J. Biol. Chem. 286, 10097–10104. 10.1074/jbc.M110.208140 21245147 PMC3060461

[B29] Seymour L. M. Jenkins C. Deutscher A. T. Raymond B. B. Padula M. P. Tacchi J. L.. (2012). Mhp182 (P102) binds fibronectin and contributes to the recruitment of plasmin(ogen) to the *Mycoplasma hyopneumoniae* cell surface. Cell. Microbiol. 14, 81–94. 10.1111/j.1462-5822.2011.01702.x 21951786

[B30] Simionatto S. Marchioro S. B. Maes D. Dellagostin O. A. (2013). *Mycoplasma hyopneumoniae*: from disease to vaccine development. Vet. Microbiol. 165, 234–242. 10.1016/j.vetmic.2013.04.019 23680109

[B31] Stemke G. W. Robertson J. A. (1982). Comparison of two methods for enumeration of mycoplasmas. J. Clin. Microbiol. 16, 959–961. 7153345 10.1128/jcm.16.5.959-961.1982 PMC272510

[B32] Thacker E. L. Minion F. C. (2010). Mycoplasmosis, in Diseases of Swine, ed Zimmerman J. (Ames, IA: Iowa State University Press), 19.

[B33] Viale M. N. Echeverria-Valencia G. Romasanta P. Laura Mon M. Fernandez M. Malchiodi E.. (2014). Description of a novel adhesin of Mycobacterium avium subsp. paratuberculosis. Biomed Res. Int. 2014:729618. 10.1155/2014/729618 25136616 PMC4130151

[B34] Voorhees R. M. Ramakrishnan V. (2013). Structural basis of the translational elongation cycle. Annu. Rev. Biochem. 82, 203–236. 10.1146/annurev-biochem-113009-092313 23746255

[B35] Wilton J. Jenkins C. Cordwell S. J. Falconer L. Minion F. C. Oneal D. C.. (2009). Mhp493 (P216) is a proteolytically processed, cilium and heparin binding protein of *Mycoplasma hyopneumoniae*. Mol. Microbiol. 71, 566–582. 10.1111/j.1365-2958.2008.06546.x 19040640

[B36] Wu Y. Jin M. Bai F. Zhang X. Hua L. Lei Z. (2012). Development and application of TaqMan-BHQ real time PCR assay for detection of *Mycoplasma hyopneumoniae* P97. Chin. Veter. Sci. 42:5.

[B37] Yu Y. Qian Y. Du D. Xu C. Dai C. Li Q.. (2016). SBP2 plays an important role in the virulence changes of different artificial mutants of Streptococcus suis. Mol. Biosyst. 12, 1948–1962. 10.1039/C6MB00059B 27077729

[B38] Zhang Q. Young T. F. Ross R. F. (1995). Identification and characterization of a *Mycoplasma hyopneumoniae* adhesin. Infect. Immun. 63, 1013–1019. 7868222 10.1128/iai.63.3.1013-1019.1995 PMC173103

[B39] Zhu W. Zhang Q. Li J. Wei Y. Cai C. Liu L.. (2017). Glyceraldehyde-3-phosphate dehydrogenase acts as an adhesin in Erysipelothrix rhusiopathiae adhesion to porcine endothelial cells and as a receptor in recruitment of host fibronectin and plasminogen. Vet. Res. 48:16. 10.1186/s13567-017-0421-x 28327178 PMC5360030

